# 527. Lower Risk of ICU Admission with Remdesivir in Patients Hospitalized with COVID-19 Pneumonia

**DOI:** 10.1093/ofid/ofab466.726

**Published:** 2021-12-04

**Authors:** Sarah Lim, Pamela Schreiner, Alan Lifson, Erica Bye, Kathryn Como-Sabetti, Ruth Lynfield, Ruth Lynfield

**Affiliations:** 1 MN Department of Health, St Paul, Minnesota; 2 University of Minnesota School of Public Health, Minneapolis, Minnesota; 3 University of Minnesota, Minneapolis, Minnesota; 4 Minnesota Department of Health, St. Paul, Minnesota

## Abstract

**Background:**

Remdesivir (RDV) was approved by FDA in October 2020 for use in hospitalized patients with COVID-19. We examined the association between RDV treatment and ICU admission in patients hospitalized with COVID-19 pneumonia requiring supplemental oxygen (but not advanced respiratory support) in MN.

**Methods:**

COVID-19-Associated Hospitalization Surveillance Network (COVID-NET) is population-based surveillance of hospitalized laboratory confirmed cases of COVID-19. We analyzed COVID-NET cases ≥18 years hospitalized between Mar 23, 2020 and Jan 23, 2021 in MN for which medical record reviews were complete. On admission, included cases had evidence of COVID-19 pneumonia on chest imaging with oxygen saturation < 94% on room air or requiring supplemental oxygen. Cases were excluded if treated with RDV after ICU admission. Multivariable logistic regression was performed to assess the association between RDV treatment and ICU admission.

**Results:**

Complete records were available for 8,666 cases (36% of admissions statewide). 1,996 cases were included in the analysis, of which 908 were treated with RDV. 83% of cases were residents of the 7-county metro area of Minneapolis-St. Paul. Mean age was 59.7 years (IQR 48-72), 55% were male, and the mean RDV treatment duration was 4.8 days (range 2-15). The proportion of cardiovascular disease (30.6% vs 23.9%, p=.003), renal disease (16.6% vs 7.6%, p< .001), and diabetes (34.7% vs 29.5%, p=0.01) was higher in the RDV untreated group, while obesity (22.3% vs 8.4%, p< .001) and dexamethasone use (54.7% vs 15%, p< .001) was more common in the RDV treated group. RDV untreated patients were more likely to be admitted to an ICU (18% vs 8.9%, p< .001) and had higher inpatient mortality than those treated with RDV (11% vs 4.4%, p< .001). After adjustment for dexamethasone use, age, sex and diabetes, treatment with RDV was associated with 48% lower odds of ICU admission (OR 0.52, 0.39-0.7, p< .001).

**Conclusion:**

We found RDV treatment associated with a significantly lower risk of ICU admission in patients admitted to hospital requiring supplemental oxygen, suggesting that treatment may prevent disease progression in this group. Further studies should assess the potential benefit of RDV combination treatment with dexamethasone.

**Disclosures:**

**Ruth Lynfield, MD**, Nothing to disclose

